# On the Power of Constructive Criticism

**DOI:** 10.1117/1.NPh.11.4.040101

**Published:** 2024-12-20

**Authors:** Anna Devor, Androu Abdalmalak, Taner Akkin, Yeka Aponte, George J. Augustine, Sabrina Brigadoi, Erin M. Buckley, Robert E. Campbell, Stefan A. Carp, Jean-Yves Chatton, Jerry L. Chen, Ji-Xin Cheng, Tomáš Cižmár, Robert J. Cooper, Ippeita Dan, Patrick J. Drew, Turgut Durduran, Valentina Emiliani, Tommaso Fellin, Amanda J. Foust, Ling Fu, Amir Gandjbakhche, Judit Gervain, Ariel Gilad, Edgar Guevara Codina, Xue Han, Elizabeth M. Hillman, Ute Hochgeschwender, Song Hu, Na Ji, Prakash Kara, Beop-Min Kim, Tiffany S. Ko, Duygu Kuzum, Jérôme Lecoq, Rickson C. Mesquita, Timothy H. Murphy, U. Valentin Nägerl, Nozomi Nishimura, Yumie Ono, Nisan Ozana, Francesco S. Pavone, Darcy S. Peterka, Ferruccio Pisanello, Anna Wang Roe, Andy Y. Shih, Shy Shoham, François St-Pierre, Sungho Tak, Lin Tian, Kai Wang, Martin Wolf, Ji Yi, Ofer Yizhar, Meryem A. Yucel

**Affiliations:** a Boston University, College of Engineering, Department of Biomedical Engineering, Boston, Massachussetts, USA, and Athinoula A. Martinos Ctr. for Biomedical Imaging, Massachusetts General Hospital, Harvard Medical School, Charlestown, Massachussetts, USA; b NIRx Medical Technologies, LLC, Orlando, Florida, USA; c University of Minnesota, College of Science & Engineering, Department of Biomedical Engineering, Twin Cities, Minnesota, USA; d National Institutes of Health, National Institute on Drug Abuse, Bethesda, Maryland, and Johns Hopkins University, Department of Neuroscience, Baltimore, Maryland, USA; e Nanyang Technological University, The Lee Kong Chian School of Medicine, Singapore; f University of Padua, Department of Developmental Psychology and Socialisation, Padua, Italy; g Emory Univ. School of Medicine, Wallace H. Coulter Department of Biomedical Engineering at Georgia Tech and Emory, Atlanta, Georgia, USA; h The University of Tokyo, Graduate School of Science, Biomolecular Chemistry Laboratory, Tokyo, Japan, and Univ. Alberta, Department of Chemistry, Alberta, Canada; i Athinoula A. Martinos Ctr. for Biomedical Imaging, Massachusetts General Hospital, Harvard Medical School, Charlestown, Massachussetts, USA; j University of Lausanne, Department of Fundamental Neurosciences, Lausanne, Switzerland; k Boston Univ., College of Engineering, Department of Biomedical Engineering, Department of Biology, Boston Massachusetts; l Boston Univ., College of Engineering, Division of Materials Science and Engineering, Department of Chemistry, Department of Physics, Neurophotonics Center, Photonics Center, Molecular Biology, Cell Biology & Biochemistry Program, Boston, Massachussetts, USA; m Leibniz Institute of Photonic Technology (IPHT) and Friedrich-Schiller University, Leibniz, Germany, and Czech Academy of Sciences, Institute of Scientific Instruments, Complex Photonics Group, Brno, Czech Republic; n University College London, Department of Medical Physics and Biomedical Engineering, London, England, UK; o Chuo University, Institute of Science and Engineering, Tokyo, Japan; p The Pennsylvania State University, Eberly College of Science, Department of Biology, University Park, Pennsylvania, USA, and The Pennsylvania State University, Huck Institutes of the Life Sciences, University Park, Pennsylvania, USA; q ICFO—The Institute of Photonic Sciences, Barcelona, Spain; r Vision Institute, Lab. de neurophysiologie et nouvelles microscopies, Paris, France; s Istituto Italiano di Tecnologia (IIT), Department of Neuroscience and Brain Technologies, Genoa, Italy; t Imperial College London, Department of Bioengineering, London, England, UK; u Huazhong University of Science and Technology, Wuhan, China; v National Institutes of Health, Bethesda, Maryland, USA; w Univ. Paris Descartes, Integrative Neuroscience and Cognition Center, Paris, France, and University of Padua, Padua, Italy, University of British Columbia, UBS Language Sciences, Vancouver, British Columbia, Canada; x The Hebrew University of Jerusalem, Faculty of Medicine, Department of Medical Neurobiology, Jerusalam, Israel; y Universidad Autónoma de San Luis Potosí, CIACYT (Coordinación para la Innovación y Aplicación de la Ciencia y la Tecnología), San Luis Potosí, Mexico; z Boston University, College of Engineering, Department of Biomedical Engineering, Boston, Massachussetts, USA; aa Columbia University, Department of Biomedical Engineering and Radiology, Mortimer B. Zuckerman Mind Brain Behavior Institute, New York, New York, USA; bb Central Michigan University, College of Medicine, Mt. Pleasant, Michigan, USA; cc Washington University in St. Louis, McKelvey School of Engineering, Department of Biomedical Engineering, St. Louis, Missouri, United States; dd University of California Berkeley, Department of Physics, Berkeley, California, United States; ee University of Minnesota, Medical School, Department of Neuroscience, Twin Cities, Minnesota, United States; ff Korea University, College of Health Sciences, School of Biomedical Engineering, Seoul, Korea; gg Children's Hospital of Philadelphia, Leonard and Madlyn Abramson Pediatric Research Center, Philadelphia, Pennsylvania; hh University of California San Diego, Jacobs School of Engineering, Department of Electrical and Computer Engineering, La Jolla, California, USA, and University of California San Diego, John Muir College, Kavli Institute for Brain and Mind, La Jolla, California, USA; ii Allen Institute for Brain Science, Seattle, Washington, USA; jj University of Birmingham, School of Computer Science, Birmingham, England, UK; kk The University of British Columbia, Faculty of Medicine, Department of Psychiatry, Vancouver, British Columbia, Canada; ll University Medical Center Göttingen, Institute of Anatomy and Cell Biology, Göttingen, Germany; mm Cornell University, Meinig School of Biomedical Engineering, Ithaca, New York, United States; nn Meiji University, School of Science and Engineering, Department of Electronics and Bioinformatics, Tokyo, Japan; oo Bar Ilan Univerisity, The Alexander Kofkin Faculty of Engineering, Department of Neuro-Engineering & Bio-Engineering, Ramat Gan, Israel; pp University of Florence, Physics Department, LENS - European Laboratory for Non-linear Spectroscopy, Florence, Italy; qq Columbia University, Zuckerman Institute, New York City, New York, USA, and Columbia University, Kavli Institute for Brain Science, New York, New York, USA; rr Istituto Italiano di Tecnologia (IIT), Center for Biomolecular Nanotechnologies, Lecce, Italy; ss Zhejiang University, Interdisciplinary Institute of Neuroscience and Technology, Hangzhou, Zhejiang, China; tt Seattle Children’s Hospital, Center for Integrative Brain Research, Center for Developmental Biology and Regenerative Medicine, Seattle, Washington, USA; uu New York University, Langone Health, Tech4Health Institute, New York, New York, USA; vv Rice University, Baylor College of Medicine, Department of Neuroscience and Department of Electrical and Computer Engineering, Houston, Texas, USA; ww Korea Basic Science Institute, Ochang, Korea; xx University of California Davis, School of Medicine, Department of Biochemistry and Molecular Medicine, Davis, California, USA; yy Chinese Academy of Sciences, Shanghai, China; zz University Hospital Zürich, Department of Neonatology, Zürich, Switzerland; aaa Johns Hopkins University, Department of Biomedical Engineering, Baltimore, Maryland, USA; bbb Weizmann Institute of Science, Department of Molecular Neuroscience, Rehovot, Israel; ccc Boston University, College of Engineering, Neurophotonics Center, Boston, Massachussetts, USA

## Abstract

The editorial discusses the practice of peer review for SPIE Neurophotonics.

As we approach the end of 2024, we thank our reviewers who take time away from their jobs, family, and friends to support non-profit publishing and ensure that novel, rigorous and impactful research appears in every issue of *Neurophotonics*. On that note, we often get inquiries from junior scientists about how to become a *Neurophotonics* reviewer and what are the criteria for good reviews. The answer to the first question is simple—publish in *Neurophotonics*! To answer the second question, let’s talk about the practice of peer review.

You, our reader, probably know that academic publishers differ in their perspectives on the anonymity of peer review. Some (e.g., *eLife*) not only reveal the reviewer identity but also publish review reports together with research articles. Others (e.g., *Frontiers*) reveal reviewers’ identity but do not publish the reports. At SPIE journals, peer review is single anonymous, i.e., the reviewer identity remains confidential.

Those in favor of full disclosure often say that it increases the quality and transparency of peer review. The reason that it works, in many cases, is that if the paper is good, reviewers’ comments would be supportive and helpful for the authors to generate a better paper. So, everyone wins. If the paper is bad, it would be rejected, and the names of reviewers would not be released, so, no harm done.

In addition, a reviewer may offer an insight that would be credited to them if the comment is published with a paper. So, it can be argued that co-publishing reviews encourages reviewers not to hold back creative ideas and interpretations.

However, those in favor of keeping confidentiality rightly point out that open criticism—fair or not—can have adverse effects on the scientific community triggering hostility, skepticism, etc. These negatives defeat the purpose, i.e., increasing the integrity of peer review.

At *Neurophotonics*, we realize this complexity and are not taking sides. As an SPIE journal, *Neurophotonics* does not reveal reviewers. Nevertheless, when you sit down to write your report, try to imagine that it would be released. Let us explain why.

Let’s say that a paper that you are reviewing is interesting and has a kind of innovation in methodology or application that you expect from *Neurophotonics* papers. You start by focusing on the big picture and summarize the strengths and weaknesses. Then, you describe what it would take, in your opinion, to address the weaknesses and make it useful for the community. How should you phrase your criticism?

In general, your role as a reviewer is to be both critical and supportive. If you place yourself on the receiving end, you as an author would like to believe that if you put in additional work that a reviewer is asking for to resolve certain issues, the reviewer would be likely to appreciate the improvement. So, when you write your review, ask yourself whether there is a path to significant improvement. If the answer is “yes,” this is where a mental exercise of imagining your report being released comes in. Do not hesitate to communicate to the authors that you support this line of research and are excited about the study. Then, encourage the authors to take a deep breath and invest more time and effort to make the best version of the paper they can. To this end, concrete guidelines to where the problems are and what can be done to strengthen the study are very helpful...when delivered without sounding dismissive or argumentative. Think of this experience as a collaboration, not a fight.

What if, in your opinion, there’s no path forward, and resolving the weaknesses would be equivalent to writing a whole new paper? In this case, this is a reject. However, do not discard your empathy tool at the door. As a reviewer, you can be equally gracious and thoughtful. Who knows, constructive criticism presented in a friendly voice may find fertile soil. Following your advice, the authors may rethink the paradigm, perform a whole new study, and then come up with an important discovery! You weren’t blamed for the reject, and it was the power of your constructive criticism that made this happen—this is a win! You won’t be credited for the discovery, either. This is a loss, but such is the duality of anonymous peer review!

Happy Holidays and Happy New Year to our authors, reviewers, readers, and SPIE friends!

*Neurophotonics* Editorial Board

**Table:** 

**Anna Devor**, Boston University, College of Engineering, Department of Biomedical Engineering, Boston, Massachussetts, USA, and Athinoula A. Martinos Ctr. for Biomedical Imaging, Massachusetts General Hospital, Harvard Medical School, Charlestown, Massachusetts, USA
**Androu Abdalmalak**, NIRx Medical Technologies, LLC, Orlando, Florida, USA
**Taner Akkin**, University of Minnesota, College of Science & Engineering, Department of Biomedical Engineering, Twin Cities, Minnesota, USA
**Yeka Aponte**, National Institutes of Health, National Institute on Drug Abuse, Bethesda, Maryland, and Johns Hopkins University, Department of Neuroscience, Baltimore, Maryland, USA
**George J. Augustine**, Nanyang Technological University, The Lee Kong Chian School of Medicine, Singapore
**Sabrina Brigadoi**, University of Padua, Department of Developmental Psychology and Socialisation, Padua, Italy
**Erin M. Buckley**, Emory Univ. School of Medicine, Wallace H. Coulter Department of Biomedical Engineering at Georgia Tech and Emory, Atlanta, Georgia, USA
**Robert E. Campbell**, The University of Tokyo, Graduate School of Science, Biomolecular Chemistry Laboratory, Tokyo, Japan, and Univ. of Alberta, Department of Chemistry, Alberta, Canada
**Stefan A. Carp**, Athinoula A. Martinos Ctr. for Biomedical Imaging, Massachusetts General Hospital, Harvard Medical School, Charlestown, Massachusetts, USA
**Jean-Yves Chatton**, University of Lausanne, Department of Fundamental Neurosciences, Lausanne, Switzerland
**Jerry L. Chen**, Boston Univ., College of Engineering, Department of Biomedical Engineering, Department of Biology, Boston Massachusetts
**Ji-Xin Cheng**, Boston Univ., College of Engineering, Division of Materials Science and Engineering, Department of Chemistry, Department of Physics, Neurophotonics Center, Photonics Center, Molecular Biology, Cell Biology & Biochemistry Program, Boston, Massachussetts, USA
**Tomáš Cižmár**, Leibniz Institute of Photonic Technology (IPHT) and Friedrich-Schiller University, Leibniz, Germany, and Czech Academy of Sciences, Institute of Scientific Instruments, Complex Photonics Group, Brno, Czech Republic
**Robert J. Cooper**, University College London, Department of Medical Physics and Biomedical Engineering, London, England, UK
**Ippeita Dan**, Chuo University, Institute of Science and Engineering, Tokyo, Japan
**Patrick J. Drew**, The Pennsylvania State University, Eberly College of Science, Department of Biology, University Park, Pennsylvania, USA, and The Pennsylvania State University, Huck Institutes of the Life Sciences, University Park, Pennsylvania, USA
**Turgut Durduran**, ICFO—The Institute of Photonic Sciences, Barcelona, Spain
**Valentina Emiliani**, Vision Institute, Lab. de neurophysiologie et nouvelles microscopies, Paris, France
**Tommaso Fellin**, Istituto Italiano di Tecnologia (IIT), Department of Neuroscience and Brain Technologies, Genoa, Italy
**Amanda J. Foust**, Imperial College London, Department of Bioengineering, London, England, UK
**Ling Fu**, Huazhong University of Science and Technology, Wuhan, China
**Amir Gandjbakhche**, National Institutes of Health, Bethesda, Maryland, USA
**Judit Gervain**, Univ. Paris Descartes, Integrative Neuroscience and Cognition Center, Paris, France, and University of Padua, Padua, Italy, University of British Columbia, UBS Language Sciences, Vancouver, British Columbia, Canada
**Ariel Gilad**, The Hebrew University of Jerusalem, Faculty of Medicine, Department of Medical Neurobiology, Jerusalem, Israel
**Edgar Guevara Codina**, Universidad Autónoma de San Luis Potosí, CIACYT (Coordinación para la Innovación y Aplicación de la Ciencia y la Tecnología), San Luis Potosí, Mexico
**Xue Han**, Boston University, College of Engineering, Department of Biomedical Engineering, Boston, Massachusetts, USA
**Elizabeth M. Hillman**, Columbia University, Department of Biomedical Engineering and Radiology, Mortimer B. Zuckerman Mind Brain Behavior Institute, New York, New York, USA
**Ute Hochgeschwender**, Central Michigan University, College of Medicine, Mt. Pleasant, Michigan, USA
**Song Hu**, Washington University in St. Louis, McKelvey School of Engineering, Department of Biomedical Engineering, St. Louis, Missouri, USA
**Na Ji**, University of California Berkeley, Department of Physics, Berkeley, California, USA
**Prakash Kara**, University of Minnesota, Medical School, Department of Neuroscience, Twin Cities, Minnesota, USA
**Beop-Min Kim**, Korea University, College of Health Sciences, School of Biomedical Engineering, Seoul, Korea
**Tiffany S. Ko**, Children’s Hospital of Philadelphia, Leonard and Madlyn Abramson Pediatric Research Center, Philadelphia, Pennsylvania
**Duygu Kuzum**, University of California San Diego, Jacobs School of Engineering, Department of Electrical and Computer Engineering, La Jolla, California, USA, and University of California San Diego, John Muir College, Kavli Institute for Brain and Mind, La Jolla, California, USA
**Jérôme Lecoq**, Allen Institute for Brain Science, Seattle, Washington, USA
**Rickson C. Mesquita**, University of Birmingham, School of Computer Science, Birmingham, England, UK
**Timothy H. Murphy**, The University of British Columbia, Faculty of Medicine, Department of Psychiatry, Vancouver, British Columbia, Canada
**U. Valentin Nägerl**, University Medical Center Göttingen, Institute of Anatomy and Cell Biology, Göttingen, Germany
**Nozomi Nishimura,** Cornell University, Meinig School of Biomedical Engineering, Ithaca, New York, USA
**Yumie Ono,** Meiji University, School of Science and Engineering, Department of Electronics and Bioinformatics, Tokyo, Japan
**Nisan Ozana,** Bar Ilan University, The Alexander Kofkin Faculty of Engineering, Department of Neuro-Engineering & Bio-Engineering, Ramat Gan, Israel
**Francesco S. Pavone**, University of Florence, Physics Department, LENS - European Laboratory for Non-linear Spectroscopy, Florence, Italy
**Darcy S. Peterka**, Columbia University, Zuckerman Institute, New York City, New York, USA, and Columbia University, Kavli Institute for Brain Science, New York, New York, USA
**Ferruccio Pisanello**, Istituto Italiano di Tecnologia (IIT), Center for Biomolecular Nanotechnologies, Lecce, Italy
**Anna Wang Roe**, Zhejiang University, Interdisciplinary Institute of Neuroscience and Technology, Hangzhou, Zhejiang, China
**Andy Y. Shih**, Seattle Children’s Hospital, Center for Integrative Brain Research, Center for Developmental Biology and Regenerative Medicine, Seattle, Washington, USA
**Shy Shoham**, New York University, Langone Health, Tech4Health Institute, New York, New York, USA
**François St-Pierre**, Rice University, Baylor College of Medicine, Department of Neuroscience and Department of Electrical and Computer Engineering, Houston, Texas, USA
**Sungho Tak**, Korea Basic Science Institute, Ochang, Korea
**Lin Tian**, University of California Davis, School of Medicine, Department of Biochemistry and Molecular Medicine, Davis, California, USA
**Kai Wang**, Chinese Academy of Sciences, Shanghai, China
**Martin Wolf**, University Hospital Zürich, Department of Neonatology, Zürich, Switzerland
**Ji Yi**, Johns Hopkins University, Department of Biomedical Engineering, Baltimore, Maryland, USA
**Ofer Yizhar**, Weizmann Institute of Science, Department of Molecular Neuroscience, Rehovot, Israel
**Meryem A. Yucel**, Boston University, College of Engineering, Neurophotonics Center, Boston, Massachusetts, USA

